# Social and cultural construction processes involved in HPV vaccine hesitancy among Chinese women: a qualitative study

**DOI:** 10.1186/s12939-019-1052-9

**Published:** 2019-09-18

**Authors:** Judy Yuen-man Siu, Timothy K. F. Fung, Leo Ho-man Leung

**Affiliations:** 1 Department of Applied Social Sciences, Faculty of Health and Social Sciences, The Hong Kong Polytechnic University, Hung Hom, Hong Kong; 2 Department of Communication Studies, School of Communication, Hong Kong Baptist University, Kowloon Tong, Hong Kong

**Keywords:** Human papillomavirus, HPV vaccination, Vaccine hesitancy, Decision making process, Women, Chinese

## Abstract

**Background:**

HPV vaccine is a prophylactic vaccine to prevent HPV infections. Recommended by the World Health Organization, this vaccine is clinically proven to be one of the most effective preventive measures against the prevalence of cervical cancer and other HPV-associated cancers and chronic genital conditions. However, its uptake rate among women in Hong Kong is insignificant—only approximately 2.9% adolescent girls and 9.7% female university students received HPV vaccination in 2014. With the notion of Critical Medical Anthropology, we aimed to identify if different influential factors, ranging from individual, societal, and cultural, are involved in the decision-making process of whether to receive HPV vaccination.

**Methods:**

We adopted a qualitative approach and conducted in-depth individual semistructured interviews with 40 women in Hong Kong between May and August 2017.

**Results:**

We noted that the following factors intertwined to influence the decision-making process: perceptions of HPV and HPV vaccine; perceived worthiness of HPV vaccines, which was in turn influenced by vaccine cost, marriage plans, and experiences of sexual activities; history of experiencing gynecological conditions, stigma associated with HPV vaccination, acquisition of information on HPV vaccines, distrust on HPV vaccines, and absence of preventive care in the healthcare practice.

**Conclusions:**

HPV vaccination is promoted in a manner that is “feminized” and “moralized” under the patriarchal value system, further imposing the burden of disease on women, and leading to health inequality of women in pursuing the vaccination as a preventive health behaviour as a result. We believe that this ultimately results in an incomplete understanding of HPV, consequently influencing the decision-making process. The “mixed-economy” medical system adopting capitalist logic also molds a weak doctor–patient relationship, leading to distrust in private practice medical system, which affects the accessibility of information regarding HPV vaccination for participants to make the decision.

## Background

HPV vaccine is a prophylactic vaccine to prevent HPV infections. Recommended by the World Health Organization (WHO) [61], it is efficacious in the prevention of infections caused by cancerous HPV strains, which can lead to cervical cancer and precancerous cervical lesions, as well as those caused by non-cancerous HPV strains, which can lead to genital warts. Receiving HPV vaccination, thus, is one of the most effective preventive measures to control the prevalence of cervical cancer and other HPV-associated cancers and chronic genital conditions in women.

Since licensure in 2006, HPV vaccines have been introduced for women in many countries and included in the immunization program of these places in order to prevent cervical cancer [40]. Australia was the first country to establish school-based HPV vaccination programs. Other early adopter countries include the United Kingdom [5, 18], the United States [41], Belgium [51], and Denmark [60]; these countries listed HPV vaccine in their national immunization schedules or national healthcare settings. On the other hand, few developing countries initially included HPV vaccines in their immunization programs considering the cost involved in vaccine delivery and competing public health priorities [25, 26]. Facilitated by scientific advances and financial assistance from civil society organizations and nongovernmental organizations, HPV vaccination has become accessible to several low- and lower–middle-income countries [30]. As of 2016, HPV vaccine has been on the national or subnational immunization schedules in > 70 countries across continents [7]. The coverage rates between high-income and low- and middle-income regions have reversed in recent years, and the implementation of national HPV vaccination programs has allowed developing regions to achieve a higher vaccination coverage than developed regions on average [7].

However, the situation in the Asian Pacific region is different from the general trend. Except for Australia, HPV vaccination coverage in this region is relatively low. In more affluent countries, such as Japan and Korea, or less affluent ones, such as Thailand and Cambodia, the HPV vaccination coverage has been approximately 10% in the current decade [38]. Hong Kong also recorded an insignificant HPV vaccine uptake rate in 2014—only approximately 2.9% adolescent girls and 9.7% female university students [14] reportedly received HPV vaccination.

The acceptability of vaccines by women is apparently influenced by various factors, such as information pertaining to [1, 29, 32] and attitude toward [6, 49] the vaccine. In Chinese communities, the low rate of HPV vaccination has been attributed to the cost of the vaccine [15], insufficient awareness regarding HPV issues [33], lack of information from primary care doctors [52], and concerns regarding the possible side-effects of the vaccine [63].

### The notion of vaccine hesitancy

It is not always straightforward for people to accept the idea of vaccination, and vaccine hesitancy [44, 45], which refers to a “delay in acceptance or refusal of vaccines despite availability of vaccination services” [62], is one of the commonly used frameworks to understand the low acceptability of vaccination. There are various ways to interpret this notion [22, 45, 47]—vaccine hesitancy is “complex and context specific, varying across time, place, and vaccines” [62]. This parlance can be further expanded by going into details about the ambiguity of this notion. Vaccine hesitancy does not indicate complete vaccine refusal, and vaccine-hesitant people are not necessarily against vaccines; rather, the notion of vaccine hesitancy focuses more on the doubt and disinclination about vaccination.

Peretti-Watel et al. [48] suggested that this notion can be understood using an explicit theoretical framework that takes some major structural features of contemporary societies into account; moreover, from a sociological perspective, two cultural features of contemporary societies seem to be involved: “risk culture” and “healthism” [3, 19]. Contemporary societies seemingly have entered a new stage that succeed the modern era or modernity. One of the significant features of late modernity basis Anthony Giddens’ formulation [19] is the post-traditional nature. Human conducts are heavily under the restraint of prescribed customs and norms, and they are therefore often done without much challenge or thought due to precedent habits and dominated traditions. Hence, the choices available to an individual are limited in a traditional society. The advancements in technology and expansion of capitalism, mass media, and industrialism across the globe have contributed to the development of the post-traditional society. This represents the growing amount of reflexivity in all domains of society from formal institutions (macro-level) to self-identity and intimacy between people (micro-level). As a result, the decline in tradition increases reflexivity for individuals in contemporary societies. Giddens [19] does not reckon this type of recession of tradition as a rejection or negation of modernity and rationality; instead, this recession of tradition configures a new stage of enlightenment that demands new knowledge and autonomy to get rid of the absolute values that command individual allegiance, thereby valuing diversity and heterogeneity. Contemporary social theorists such as Jürgen Habermas (modernization and the colonization of lifeworld by the system) and Alain Touraine (post-industrial society and new social movements) concur to this type of idea. In particular, Giddens [19] related his ideas of the reflexive age of late modernity to the risk society. Coupled with the rise of distinct sets of values, aspirations, and expectations, reflexive modernity also resulted in awareness of risk, vulnerability, and insecurity. It is the consequence of the process of making tradition enfeeble and reconstructing the aspirations of modernity. The vaccination-associated matter is therefore affected by this feature of contemporary societies [48]. The value challenged under contemporary societies also applies to science and medicine. The doubts and hesitations revolving vaccination therefore stem from the process of reconstructing values and knowledge.

Based on these theoretical viewpoints, vaccine hesitancy should not be interpreted as merely a behavioural outcome; it is more appropriate to consider it as a decision-making process [39, 48], since the decision of whether to get vaccinated does not necessarily represent the views of an individual on vaccination, but it is rather an empowering process that involves questioning and assessing the risk of different health issues. The convolution of the notion of vaccine hesitancy is based on that its focus should not only be put on the decisions made by an individual but also on the factors influencing the decision-making process. It is not merely about why people refuse to get vaccinated; rather, we should examine what makes people resist or accept vaccination.

The Strategic Advisory Group of Experts [50], part of the WHO, formed a working group on immunization and vaccine uptake in order to tackle vaccine hesitancy. They proposed the “three Cs model.” The three Cs represent confidence, complacency, and convenience, which are the three core determinants that affect the decision-making process regarding vaccination. Confidence in this model refers to trust in the effectiveness and safety of vaccines, the system delivering vaccines, and the motivations of policymakers deciding on the needed vaccines [50]. Vaccine complacency comes to existence when the perceived risks of vaccine-preventable conditions become low. Because perceived risks are low, vaccination no longer appears to be a necessary preventative action [50]. Convenience describes the effect that the physical availability of vaccines, affordability, willingness-to-pay, and ability to understand may have on vaccine hesitancy [50]. Based on this tripartite model, it is suggested that the decision of whether to get vaccinated is related to our subjective perception. SAGE [50] published a matrix categorizing factors that influence these three decisive elements into three groups. This vaccine hesitancy matrix indicates that various factors, ranging from the micro/personal level to macro/societal level, affect the decision-making process regarding vaccination. Coupled with the wide range of influential factors, the entire process of vaccine hesitancy should be recognized as a dialectic one that is manipulated by multidimensional levels of elements from the society, culture, economy, politics, personal awareness, and literacy and vaccine-related matters.

### Significance

The formulation of vaccine hesitancy suggested by SAGE offers a tripartite model (confidence, complacency, and convenience) to understand core determinants affecting the decision-making process regarding HPV vaccination. Although the “three Cs model” suggests that vaccination hesitancy should be interpreted as a decision-making process, it is a summary model without delineating the interaction of different individual, social, and cultural factors. This paves the gap of the vaccination studies in current literature. Health Belief Model and Theory of Planned Behavior are the most popular behavioral health theories that have been used in the vaccination studies [9, 17]. However, these two theories focus more on the micro aspects of individual reasons and fail to investigate the social and cultural factors that affect individuals’ decision-making process. In view of this literature gap, Critical Medical Anthropology (CMA) as suggested by Baer, Singer, and Susser [2] is adopted in this study to provide a more holistic investigation in this decision-making process. CMA suggests that cultural system and the macro-social systems that are mentioned in the four social-level analysis can also be influential in one’s health behaviour. According to CMA, a person’s health behaviour and perception are to be influenced by four social levels—from the individual, micro-social, intermediate-social to macro-social (Baer, Singer, & Susser, 1997). At the individual level, personal factors and social support network influence a person’s health behaviour and perception (Baer, Singer, & Susser, 1997). On the micro-social level, interaction between a person and healthcare providers is believed to influence the person’s health behaviour (Baer, Singer, & Susser, 1997). For the intermediate-social and macro-social levels, policy, ideology, ethno and religious beliefs, and cultural values are at work to influence a person’s health behaviour and perception [2]. Furthermore, past studies note that sexual values and stigma on the HPV vaccine can lead to people’s hesitation in receiving HPV vaccination [52–54]. This article therefore adopts the CMA framework to provide a holistic investigation on how the interacting four social levels affect the decision-making process of HPV vaccination.

The HPV vaccination rate has been low among women in Hong Kong [14], and during the period of this study, there was a lack of institutional support to women for HPV vaccination in Hong Kong: these factors made Hong Kong an interesting region to conduct this study as the decision-making process was assumed to be without any institutional intervention. This study investigates the decision-making process of HPV (non) vaccination among Chinese women in Hong Kong; it does not merely investigate the barriers that make women reluctant to HPV vaccination, but it also examines factors that motivate them to get vaccinated. Therefore, this study aims at examining these factors underlying the decision making of HPV vaccination, and how these factors are interocking with the healthcare system of a place.

## Methods

### Data collection

We adopted a qualitative approach to conduct an in-depth investigation of elements that influence the decision-making process of women regarding HPV (non) vaccination in Hong Kong. Individual semistructured interviews were conducted to gain an in-depth understanding of the complex social and cultural processes that govern this decision.

Forty female participants were interviewed between May and August 2017. Purposive sampling was employed for participant recruitment, with the following sampling criteria: (1) women aged 18–59 years at the time of the study, (2) lived and received education in Hong Kong since birth, and (3) Hong Kong Chinese in ethnicity. HPV vaccination is suggested for women aged ≥9 years in Hong Kong [12]; however, we excluded women aged 9–17 years as children and adolescents within this age group are expected to be under parental influence and have limited autonomy in making decisions pertaining to vaccination. We therefore purposively sampled women aged ≥18 years at the time of this study. Furthermore, only those who had lived and received education in Hong Kong since birth and who were Hong Kong Chinese were sampled to ensure the participants had an intense social and cultural experience, including vaccination experience, in Hong Kong. This could avoid the influence of social and cultural systems from other communities that may affect the participants’ health perceptions and behaviour. Those who have received and not yet received HPV vaccination were eligible to be recruited.

The participant recruitment process was conducted in two stages. First, posters were put up in public facilities in a university campus in April 2017, which resulted in the successful recruitment of 10 participants. The majority of them were < 30 years of age and had received HPV vaccination at the time of the study. Second, to ensure a wider sociodemographic mix of participants, the next round of recruitment was conducted in the community, resulting in the recruitment of 30 women. Posters were put up in a primary care clinic at a residential new town—Tseung Kwan O—from May to June 2017, targeting women who were aged ≥30 years. Among all the residential new towns in Hong Kong, Tseung Kwan O had the third highest population, with the third highest percentage of population having attained postsecondary education, and with the second highest labor force participation rate and median monthly income [10]. By involving this second sampling site, we expected to investigate perceptual, social, and cultural elements that influence the decision-making process of women regarding HPV (non)vaccination—this could be facilitated keeping financial factors aside, considering that this sampling site had a relatively high number of residents with postsecondary education and a relatively high monthly income. Participant recruitment was considered to be complete after data saturation. In total, we purposively sampled 40 women aged 18–58 years. Participant characteristics are shown in Table 1.

**Table 1:** Characteristics of informants (*N* = 40)

Characteristics	Percentage (%)	Number of People
Gender		
Female	100	40
Age		
18–20	12.5	5
21–25	25	10
26–30	12.5	5
31–35	15	6
36–40	12.5	5
41–45	7.5	3
46–59	15	6
Marital Status		
Single	57.5	23
Married	32.5	13
Others	10	4
Whether have gotten HPV vaccines		
Yes	30	12
No	70	28
Whether have gynaecological screening regularly		
Yes	32.5	13
No	67.5	27
Whether have children		
Yes	32.5	13
No	67.5	27

The interviews were open-ended to offer flexibility to participants in expressing their perceptions and experiences. Prior to the interview, an interview question guide (see Appendix) was prepared, keeping past literature on factors that affect HPV vaccination among women as a reference [27, 28, 31, 52, 53]. The guide included specific questions, while maintaining flexibility, and contained three parts. The first part focused on investigating the knowledge and feelings of women about HPV vaccination and HPV-associated issues. The second part aimed to investigate factors that motivated or discouraged the decision of women regarding HPV (non)vaccination. Considering that not all women had received HPV vaccination, we implemented two strategies. For HPV-vaccinated women, the aforementioned two parts gained information on their experience of getting vaccinated and what motivated their decision, respectively. On the contrary, for non-vaccinated women, the second part of the interview aimed to investigate the barriers that led to non-vaccination and also their concerns regarding non-acceptance of HPV vaccination. The third and final part of the interview aimed to identify the possible elements that encouraged them to get vaccinated. The interviews were conducted using the question guide in a semistructured but an open-ended format—this ensured that interviews were solely focused on the research topics while providing flexibility to all participants in freely expressing their opinions. This in turn allowed us to gain an in-depth understanding of their subjective thoughts.

The interviews were conducted between May and August 2017, taken place in a private room of the first author’s institution to ensure participants’ privacy. The first author was the interviewer for all 40 interviews. As the gender of the interviewer was the same as of the participants, the sensitivity and reactivity of participants was lower given the sensitivity of the topic [35]. Moreover, having the same interviewer conduct all 40 interviews resulted in enhanced consistency and reduced the risk of data flaw. Throughout the interview process, the interviewer constantly probed regarding factors from various aspects that could influence the decision-making process of women. Each interview lasted for 60–90 min and were conducted in Cantonese Chinese, the native dialect for both the interviewer and participants. As there was no language barrier, an in-depth discussion could take place between the interviewer and participants. No participants dropped out from the study. After interview completion, each participant received supermarket cash coupons worth HK$200 to acknowledge their participation.

### Ethics approval

We received ethical approval from the Committee on the Use of Human and Animal Subjects in Teaching and Research of Hong Kong Baptist University (no. HASC/15–16/0047). Prior to the interview, all participants were given an information sheet and consent form in their mother tongue language. The interviewer answered and clarified all enquiries from participants. Written consent and permission to audio-record the interviews were obtained from all participants. All interviews were anonymously conducted to ensure confidentiality. Each participant was assigned a code and a pseudonym in the data for confidentiality purposes. To further protect the privacy of all participants, all audio files were destroyed after transcribing and accuracy checking. All data were stored in password-protected files/folders and were only accessible to research team members.

### Data analysis

All interviews were transcribed verbatim. Post-transcription, member checking was performed and each manuscript was checked by the participant to ensure zero content distortion. The transcripts were then translated from Chinese to English, and backtranslation was conducted to ensure that the transcribed interviews were not distorted. The data were formatted in a common format and a backup of each file was prepared. The first and third authors conducted the coding process individually to analyze the data; we used an inductive coding strategy to identify the subjective thinking and behavioural patterns of all participants [34]. The transcripts were analyzed line-by-line. The raw interview texts were thoroughly read for content familiarization and then re-read to determine possible themes [58]. Distinct concepts were developed and used in memo documentation to enable systematic analysis of interviews. The transcripts were segmented into meaning units, which were labelled and then collapsed into categories [58]. Categories and themes were created from actual phrases in specific text segments. Upper-level categories were identified based on the research questions, and in vivo coding was conducted [58]. Recurrent categories were highlighted. Overlapping codes and categories were consolidated to form broader themes after repeated examination and comparison [58]. The codes, categories, and themes derived from the data, alongside supporting interview quotes, were documented in a coding table [21], where designated concepts and categories were highlighted to translate the interviews into meaningful symbols to enable understanding of the thoughts of all participants. As the coding process was separately conducted by the first and third authors, we noted some overlapping and redundancy among the categories. The category system was thus further refined to reduce verbosity among the categories, and therefore, the most meaningful themes were figured out. Consensus in the coded data was achieved. NVivo 11 was used for coding and analytical processes. Data saturation, which was defined as the point at which no new themes were to emerge from the data [34], was thus achieved.

## Results

The rationale behind the decision-making process of participants regarding HPV (non) vaccination involved a combination of individual, social and cultural factors at four social levels according to Baer, Singer, and Susser [2]. Analysis of interview data showed the following themes were at work: perceptions of HPV and HPV vaccine; perceived worthiness of HPV vaccine, which was affected by its cost, marriage plans, and experiences of sexual activities; history of experiencing gynecological conditions; stigma associated with HPV vaccination; acquisition of information on HPV vaccines; distrust on HPV vaccines; and absence of preventive care in the healthcare practice.

### Individual level

#### Perceptions of HPV and HPV vaccine

The participants’ perceptions on HPV and HPV vaccine could influence their health behaviour and their decision-making process. All participants had some understanding about the relationship between HPV and cervical cancer and about HPV vaccination being a preventive measure for cervical cancer. Most participants believed that HPV vaccination could only confer protection against cervical cancer:

*We [my colleagues and I] saw an advertisement about the cervical cancer vaccine. It is said that if we want to protect ourselves from cervical cancer, then we should get the jabs. The advertisement just mentions cervical cancer … It is an advertisement for cervical cancer. I don’t know if the vaccine can prevent other diseases. [Participant 18]*

Only a few participants knew that HPV can lead to genital warts; however, they rarely knew that HPV vaccination can also help prevent genital warts:

*HPV mainly results in cervical cancer? And other diseases such as … hmm … I am not sure … but maybe something related to the sexual organ, like [genital] warts. I have heard about HPV vaccination; it is for the prevention of cervical cancer. [Participant 6]*

The most prominent understanding among participants was that HPV vaccination can prevent cervical cancer; most were unaware of the other uses of HPV vaccine, although they knew that the vaccine has other preventive uses:

*I know that the vaccine can prevent cervical cancer and hmm … some kinds of viruses … I remember that I once got a leaflet stating that the vaccine can prevent some diseases … and cervical cancer. Sorry, I can recall cervical cancer only. [Participant 1]*

In most cases, participants called HPV vaccine “cervical cancer vaccine.” This “nickname” made them believe that the vaccine is exclusively for women and only for the prevention of cervical cancer:

*When I saw the information about the vaccine, I thought that it is great to have a new way to protect women. I think the vaccine is for women only, because the vaccine is a cervical cancer vaccine, so it prevents cervical cancer. Only women will suffer from it. Men will never have this disease. [Participant 37]*

The Chinese naming of HPV vaccine thus led participants to focus on its efficacy against cervical cancer only and overlook its other preventive uses. In addition, female celebrities involved in promoting the vaccine via advertisements, which most participants found impressive, further presented the vaccine as being exclusively for women:

*There are two brands of HPV vaccines. One has Ah Sa [a female Hong Kong singer] as the spokesperson, and the other has GEM [a female Hong Kong singer] as the spokesperson. We [my friends and I] call those vaccines as “Ah-Sa-vaccine” and “Ah-GEM-vaccine”. I think this vaccine is clearly for women only, because Ah Sa and GEM are women. [Participant 4]*

Besides the relationship between HPV and cervical cancer being the most prominent understanding among participants, the relationship between HPV and AIDS was also a prominent stereotype. HIV is literally similar to HPV, which seemed to confuse some participants as they misinterpreted HPV as HIV:

*HPV? Is it the cause of AIDS? I remember that AIDS is caused by a virus that has these 3 alphabets. [Participant 35]*

#### Perceived worthiness of HPV vaccine

##### Cost

Although all participants had good knowledge about the preventive use of HPV vaccine against cervical cancer, not many non-vaccinated participants were motivated to get vaccinated. One of the barriers was the cost of the vaccine, in particular the high cost of the vaccine failed to make the vaccine to become prioritized in participants’ thoughts:

*I could get a discount [for the HPV vaccine] when I was studying at university. However, I didn’t have a stable income at that time, so I did not get vaccinated. It doesn’t mean that I didn’t want to get the vaccine, but just that I didn’t want to spend such a large amount of money on the vaccine at that time. It was more sensible to spend those thousands on other more important things at that time. Getting vaccinated was not a priority for me at that time. [Participant 7]*

It appeared that if the vaccine was offered at a discounted cost, participants had a higher incentive to get vaccinated and were thus more motivated:

*I took the jabs when I was a final year [undergraduate] student. There was a promotion booth [of HPV vaccine] in the campus … It was pretty expensive, but the cost had already been discounted. It would be even more expensive if I took the jabs outside the campus after I had graduated. [Participant 4]*

On the other hand, price of the HPV vaccine can be varied in different private practice healthcare providers, and some participants were suspicious about the varied costs of the vaccines, making them hesitant to get vaccinated:

*I wanted to get the vaccine before, so I asked [the clinic] about the cost [of the HPV vaccine]. I was told that there was a vaccine which could prevent 9 types of (HPV) viruses, costing around 2,000 dollars. I thought the cost was okay. But later when I asked [about the vaccine] again, the clinic told me that there was a new [HPV] vaccine which could provide more protection but would cost around 4,000 dollars. It was really expensive … I wondered whether launching a new vaccine is a way for pharmaceutical companies to make profit. Finally, I did not take the vaccine. [Participant 35]*

##### Marriage plan

Although HPV vaccines are available on a self-pay basis to most middle- and high-income female population in Hong Kong, the cost alone cannot completely explain the decision-making process—to participants, the cost did not just imply how much would they have to spend, but it also referred to whether the vaccine was worth getting at all. Such an assessment of worthiness was in fact a complex process: it involved the perceived efficacy of the vaccine by participants in relation to their risk perception of developing cervical cancer and/or other HPV-associated conditions (if the participant could recognize those possible conditions). The perceived worthiness of the vaccine was correlated with marriage plans:

*I read the advertisement of HPV vaccination... I know that the vaccine can prevent cervical cancer. However, I don’t think I have an urgent need to get the vaccine, because I still do not have any plans to get married. There is no use to get vaccinated if you are not getting married. I may consider getting vaccinated if I am going to get married. [Participant 24]*

Non-vaccinated participants perceived the plan of getting married as remarkable to make the vaccine worthy for them. The perceived worthiness of the HPV vaccine was also related to the perceptions of participants about its protective value:

*I know the vaccine can prevent cervical cancer. However, I also know that there [the vaccine] is no 100% guarantee [for the efficacy]. I still have the chance of getting cervical cancer even after getting vaccinated. Then what is the point in spending such a large sum of money for a vaccine that cannot provide full protection to me? [Participant 25]*

The cost of the vaccine appeared to be a remarkable factor in the decision-making process. However, in addition to the actual monetary value of the vaccine, cost consideration was also based on the perceived worthiness of the vaccine, which was majorly influenced by the risk perception of developing cervical cancer—related to marriage plans in most circumstances—and/or other HPV-associated conditions as well as the perceived protective value of the vaccine.

##### Experiences of sexual activities

Sexual experiences and frequency of sexual activities were closely related to the perceptions of participants about the worthiness of getting vaccinated for HPV and thus played a role in their decision-making process. The more their sexual experiences and frequency of sexual activities, the higher was the perceived risk of having cervical cancer. With regard to the understanding of participants, HPV-related risk implied the risk of having cervical cancer, not other HPV-associated conditions. The following perceptions were frequently mentioned in the interviews:

*The advertisement said that all women need the vaccine … But as I know, this virus is sexually transmitted, so I think only those who have many sexual partners and frequent sexual activities are at a higher risk … I don’t think I am at a high risk; I am not that kind of person, so I don’t think I need the vaccine. [Participant 7]*

*Sex is a way of transmitting HPV. If people engage in sexual activities more and if they have many sexual partners, they will have a higher chance of getting the virus and related diseases for sure. Also, sex can hurt the womb. More sex more harm … For those who do not indulge in sexual activities often or have just one sexual partner, I do not think they need the vaccine. [Participant 25]*

Hence, participants believed that there exists a close relationship between sexual activities and cervical cancer incidence. Most participants had the perceptions that HPV, and thus cervical cancer, is sexually transmitted. They commonly possessed a stereotype of being promiscuous, which, as per the participants, referred to having more than one sexual partner and indulging in frequent sexual activities. This in turn would be harmful to the uterus, increasing the chances of developing cervical cancer. As all non-vaccinated participants expressed that they were either abstinent or only engaged in sexual activities with one stable partner, they perceived themselves as being at a low risk of cervical cancer; consequently, they believed that there was no real need of being vaccinated for HPV.

Sexual experiences were a key determining factor in the decision-making process. The participants believed that sexual experiences affect the efficacy of HPV vaccine, and this discouraged them to get vaccinated:

*When I was about to take the first dose, the nurse said that the efficacy of the vaccine is better for those who have never had any sexual experience. As I have had no sexual experiences, I think I can get the most from the vaccine and the vaccine is worthy. However, some of my friends, after whispering to one another, decided not to get the vaccine; I guess they must have had sex before, and getting the jab would be a waste. [Participant 1]*

The pattern and frequency of sexual activities were thus interlinked with the perceived worthiness of getting vaccinated for HPV; these were the other key determinants affecting the decision-making process. The perceptions about sexual activities in relation to HPV vaccines as possessed by the participants, however, were to some extent contradictory. On one hand, having no sexual experience was believed to enhance the efficacy of HPV vaccine, whereas on the other, no sexual experience also reduces the risk of getting cervical cancer and thus the perceived need of getting vaccinated.

#### History of experiencing gynecological conditions

Another factor influencing the decision-making process was the history of experiencing gynecological conditions. Participants with a history of cancer or other physical conditions that are exclusively observed in women were noted to have a higher perceived need of getting vaccinated for HPV. For such participants, the nature of gynecological conditions, such as menstruation-related conditions, was similar to cervical cancer. They often interpreted gynecological conditions as the insufficiency of the uterus, and such “uterus insufficiency” enhanced their sense of awareness and vulnerability toward cervical cancer, remarkably affecting their decision-making process and serving as a motivation to get vaccinated:

*Irregular menstruation is a symptom showing that the uterus is weak. This indicates that the uterus is not strong enough to fight against the disease [cervical cancer]. Therefore, it is better to get vaccinated if you have menstruation problems. [Participant 40]*

The presence of gynecological conditions in the family and social network of participants was also closely related to their awareness and perceived risk of getting cervical cancer—such a presence motivated them to think about getting vaccinated:

*Having uterus-related problems is not rare in my family. I have three elder sisters and two of them suffer from [uterine] fibroids. Their daughters have [uterine] fibroids as well … We [my sisters and nieces] all concern about our uteruses, so we used to talk about the [HPV] vaccine. We have been thinking if the [HPV] vaccine can provide more protection to us … My sisters [who suffer from uterine fibroids] have had their uterus removed already, and my nieces got the [HPV] vaccine soon afterwards [after we have discussed the efficacy of the vaccine], hoping that the vaccine can provide more protection to them. [Participant 26]*

Besides the family members of participants, the experience of people getting cancer, particularly cervical cancer, in their social network also enhanced their awareness of HPV vaccination. Such experiences by those in their social network familiarized participants with cervical cancer, serving as a determinant in their decision-making process:

*I did not take the cervical cancer vaccine in the past. However, many of my friends have been suffering from cervical cancer in recent years. I know them, and I know they are well-behaved and have led a healthy lifestyle. I did not expect them to have cervical cancer. My friends’ experiences have motivated me to take the vaccine now, because cervical cancer can happen to good women as well. [Participant 9]*

However, in a few cases, the experiences of family members and those in the social network also had a negative influence on the decision-making process of a few participants, which in turn reduced their motivation to get vaccinated:

*Everyone can have cancer in any part of the body … so there is no need to take any special preventive measures against cervical cancer. To prevent cervical cancer, or I should say all types of cancer, I still believe we should use a more holistic approach to enhance our health … I live in a healthy way, so I don’t think I need the [HPV] vaccine. If I have been leading a healthy lifestyle but still I get that cancer [cervical cancer], then that is fate. You can do nothing to change your fate; even if you can save yourself from cervical cancer by getting vaccinated, you will still suffer from other cancers. [Participant 29]*

In contrast, the absence of gynecological conditions in the family and social network of participants, particularly in case of non-vaccinated participants, resulted in unawareness among them regarding the risk of cervical cancer and the need of getting vaccinated:

*I don’t think I am at a high risk of getting cervical cancer … None of my family members have cervical cancer, so I suppose I also have a very low chance of having it. Therefore, I don’t think I need to get vaccinated. [Participant 3]*

Thus, the decision-making process regarding HPV vaccination was influenced if participants themselves or people in their social network experienced gynecological conditions. Such experiences familiarized participants with cervical cancer, enhancing their awareness and perceived risk of cervical cancer, and eventually their motivation to get vaccinated.

### Micro-social level

#### Acquisition of information on HPV vaccines

The acquisition of information on HPV vaccines was crucial to the decision-making process. Sufficient information regarding the vaccine apparently served as an important motivation factor for most participants:

*I chose that one [vaccine] with Ah Sa [female celebrity] as the spokesperson, because the clinic provided more information about that vaccine than the other one [vaccine] with GEM as the spokesperson. [Participant 5]*

On the other hand, difficulty in obtaining information and confusing information discouraged participants:

*I have been thinking about taking the vaccine. There are 9-in-1, 6-in-1, and 4-in-1 vaccines, but the information on these vaccines is rare and too confusing. I don’t really know the concrete difference among the three vaccines, and it is difficult to obtain more information. I think I will need to obtain more information before I can decide whether I would want to get vaccinated, and if so, which one I would choose. I don’t know where to get more information, so it is difficult for me to decide. [Participant 14]*

The efficacy and side effects of HPV vaccine were the most needed information that could influence the decision-making process of participants. Merely knowing its efficacy against cervical cancer was far from adequate for most participants. Rather, the eligibility to get vaccinated, duration of efficacy, and conditions that the vaccine could prevent were key insights that participants sought; such information was however seldom available:

*I know that the cervical cancer vaccine can prevent against cervical cancer, but I do not know if it is okay for me to get the jab. I suspect I am too old to get it. [She was 56 years old.] I have tried to search for the answer but failed. What I know is that there is a vaccine called cervical cancer vaccine from the leaflets and bulletins that I got from clinics, hospitals, or even from The Family Planning Association of Hong Kong [a non-government organization in Hong Kong providing medical and counselling service in sexual and reproductive health]. That’s all what I know about this vaccine. No one has told me whether I am eligible to get the jab. [Participant 36]*

### Intermediate-social level

#### Absence of preventive care in the healthcare practice

Few participants had attempted to seek more information about the vaccine from their doctors, but they often failed to obtain any robust information. This negatively influenced their decision-making process:

*Indeed, I want to ask the doctor about the cervical cancer vaccine. However, the consultation is too rushed. There is no time for me to ask any questions. Similar to most typical consultation scenarios that you can imagine, the doctor just asked me when I got sick and the symptoms, and then simply gave me some medicines and asked me to leave. [Participant 8]*

Lack of explanation by healthcare providers also served as a barrier:

*I have asked the doctor once in the past whether I need to get the jab. He responded by asking me how old I was, and then he said I didn’t need the jab. That’s all! The doctor didn’t explain anything to me. Is it because I am too old for the vaccine or could there be other reasons? I have no idea because the doctor did not explain anything. [Participant 17]*

Consultation with doctors in Hong Kong primarily involves discussions on curative and treatment methods, rather than on preventive measures. People visit doctors mainly when they are sick. Doctors thus do not show much enthusiasm in dealing with inquiries pertaining to vaccines. Moreover, several participants fail to realize that can in fact discuss issues pertaining to vaccination with doctors:

*I have many questions about the vaccine, but didn’t realize that I could ask my doctor. When you go to see a doctor, you are sick. Therefore, I don’t want to be in the clinic for long. I just want to see the doctor for my sickness, have it dealt with, and then leave as soon as possible. I have never thought of asking doctors about vaccines, because I go to them for getting treated, not for discussing vaccination. [Participant 25]*

Vaccination is a preventive intervention. In the typical clinical practice in Hong Kong, it is unusual for patients and doctors to discuss preventive care, such as vaccination, within the premises of consultation. A few participants still did mention that their doctors suggested them to get vaccinated against HPV as part of consultation; this however made participants suspicious of the intention of their doctors:

*The doctor has never mentioned this issue [HPV vaccination] in the past consultations. I didn’t ask him. However, he suddenly suggested the vaccine to me … I was a bit shocked and wondered if he wanted to make more money by selling the vaccine to me. [Participant 28]*

Although some participants already had an established relationship with their doctors, the suggestion of vaccination, in most cases, made them suspicious of the intention of their doctors:

*My gynecologist had suggested me to take the vaccination. However, I do not think I have any such need because the pap smear result was normal at that time, so why do I need to take the vaccine? After the doctor informed me that my pap smear result was normal, he then went on to ask me if I wanted to take the jab. His tone, facial expression, and gesture were quite suspicious to me. He smiled maliciously, making it seem as if he was selling the vaccine to me rather than giving professional advice. [Participant 36]*

HPV vaccines in Hong Kong are mostly available on a self-pay basis and administered by private practice doctors. As preventive care is not a clinical norm in Hong Kong, doctors suggesting vaccination to patients is sort of exceptional; such advice can in fact lead to doubt and suspicion among patients. Also, as stated by participants, they had no intention to ask their doctors about getting vaccinated because the stereotypical linkage among doctors, sickness, and treatment is much stronger than the linkage between doctors and preventive care. Thus, if vaccination was recommended by doctors, participants became suspicious and assumed that doctors had commercial intentions. Such social norms in clinical practice thus negatively influenced the decision-making process of participants.

The standpoint of government health authorities about HPV vaccines, on the other hand, had a remarkable positive influence on the decision-making process. Participants perceived the information provided by the government as more credible and reliable than that provided by their doctors:

*If it is an important vaccine for women, why doesn’t the government enforce all women to take it? Yes, if the government takes a more active role in promoting the cervical cancer vaccine, I will think it [vaccination] is important and urgent. [Participant 32]*

From an overall perspective, information pertaining to HPV vaccination was a significant factor facilitating the decision-making process. However, the information had to be perceived as trustable and credible. Unfortunately, such information was limited, constraining the decision-making process of participants.

### Macro-social level

#### Stigma associated with HPV vaccination

Promiscuity was commonly perceived by participants as an important cause of cervical cancer. They believed that everyone did not need HPV vaccination; rather, sex workers and promiscuous people with more than one sexual partners were perceived as having a higher need to get vaccinated. Considering the stereotypical relationship between cervical cancer and sexual activity, getting vaccinated for HPV was beyond a health issue to participants—it was more of a moral issue to them:

*The moral standard in our society is not good already. I feel some girls may think that being vaccinated will allow them to have sex more freely and openly. They would think they can do whatever they want after getting vaccinated. I think this is not good. Education is needed for these girls to have a correct attitude toward sex. They won’t care about these [moral] issues if they know that they are totally safe from cervical cancer after the vaccination. [Participant 23]*

Consequently, some participants were not in the favor of getting vaccinated:

*Probably one of the negative consequences of promoting cervical cancer vaccination is that girls may think that “I won’t get cervical cancer anymore,” so they have nothing to fear and may have casual sex with others more freely. [Participant 26]*

Thus, HPV vaccine was perceived as a facilitating agent that could encourage immoral, promiscuous, and unsafe sexual behaviour in vaccinated women, as they would no longer fear cervical cancer. This perception also represents a stereotype in vaccinated women who are to be perceived as violators of the moral value system. Such perception indicates that the cultural meanings of immorality and promiscuity in the context of HPV vaccines influence the decision-making process of participants. Such a cultural stereotype did not merely influence the need to get vaccinated, but it also intensified the moral burden on those who did get vaccinated.

#### Distrust on HPV vaccine

Our study participants were also majorly concerned about the side-effects of HPV vaccine. Some were worried because of the first-hand experiences of people in their social network, but most were worried due to news reports on the side-effects of HPV vaccines, which they often obtained from the Internet. Such news reports remarkably served as a barrier and negatively influenced the decision-making process:

*I have read some news on the Internet reporting the serious side-effects of getting vaccinated. I’m really worried if the vaccine is safe or not, so I still have not made up my mind. I want to wait to see if there are any side-effects being reported … I cannot remember [the side-effects] exactly. I just have heard that some people died after getting vaccinated, while others have gotten paralyzed. I remember these side-effects happened overseas, but they sound horrible, so I dare not get the vaccine … I think the news is trustable. After all, I do not think doctors or drug companies [pharmaceutical companies] will inform you of these side-effects because they are selling the vaccine. [Participant 37]*

Most participants were not exactly aware of the side-effects of HPV vaccines; they still stereotyped the vaccine as being able to cause death and paralysis.

## Discussion

As demonstrated by our study participants, the following factors intertwined to influence their decision-making process: perceptions of HPV and HPV vaccine; perceived worthiness of HPV vaccine, which was affected by its cost, marriage plans, and experiences of sexual activities; history of experiencing gynecological conditions; stigma associated with HPV vaccination; acquisition of information on HPV vaccines; distrust on HPV vaccines; and absence of preventive care in the healthcare practice. These factors involve the complex and interlocking relationship of the four social levels.

In sociology, meanings are embedded in the institutional fabric of society, so reality, or more specifically the understanding of reality, is recognized as a result of social processes of meaning construction and interpretation [4]. What people reckon as definite and take for granted is cultivated from interactions among themselves and structures, which is the idea of social constructionism. Social constructionism purports that our beliefs, ways of thinking, and values are not inherently, innately, or objectively given, but are constructed within the framework of social interaction with others. Reality and knowledge defy objectification, but are, rather, “a linguistic creation that arises in the domain of social interchange.” Such a process of social construction could be observed with regard to HPV vaccines—the understanding of our study participants regarding the vaccine was related to the social processes of feminization and moralization.

### Feminization of HPV vaccine

HPV vaccination is an effective preventive measure against cervical cancer. In addition, HPV vaccine can prevent other types of anogenital cancers and other HPV-associated conditions such as genital warts. Genital warts are indeed the commonest outcome of HPV infection [11]. In the United States, nearly 12,000 women are diagnosed with cervical cancer every year; but there are approximately 340,000 to 360,000 women and men being affected by genital warts every year [11]. However, our findings still indicated that the promulgation of HPV vaccination has been over-emphasizing the competency against cervical cancer. The additional benefit against genital warts has been largely underplayed in the blurb of the vaccine. As a result, prevention against cervical cancer was found to be a popular perception of efficacy among all participants, and they seemed unaware of the other preventive effects of HPV vaccine. This could be attributed to our study participants referring that HPV vaccine as “cervical cancer vaccine,” and this “nickname” made them assume that the vaccine is exclusively for the prevention of cervical cancer and for women, thereby feminizing HPV vaccine.

According to the Sapir–Whorf hypothesis, language and vocabulary can influence our perceptions [57]. The “real world” for us is unconsciously constructed based on the language of our social network. In other words, language contributes toward creating reality, shaping our worldview [57] and consequently affecting our perceptions and behaviours. The belief of our study participants that HPV vaccination—“cervical cancer vaccine”—is primarily effective to avert the incidence of cervical cancer alluded that the vaccination is exclusively for women, symbolizing language acts as the social construction process of feminization of HPV vaccines.

Social constructionism purports that the so-called reality is maintained by social and cultural systems, with its embodiment embedded in the continuing human activities and communication among people [4, 8, 36, 59]. The representation of reality is a projection of societal and cultural values. Accordingly, the feminization of HPV vaccines should be viewed as a revelation of certain institutional values. The imbalanced degree of emphasis on the efficacy of HPV vaccines against cervical cancer and other HPV-associated conditions has resulted in an imbalance in the promotion of HPV prevention, presenting women as being more vulnerable to HPV than men in the social discourse.

Gender studies have indicated that differences between men and women not only pertain to biological sexual differences but also to hierarchical differences in terms of intuitive, ontological security, and power [20]. Gender values in societies are also reflected in healthcare. The difference in power between women and men in patriarchal societies are tightly related to the social construction of illness, in which women are presented as a “diseased body” and as weaker than men that requires more biomedical intervention [37], consequently resulting in victimization. The Chinese “nickname” for HPV vaccine, i.e., “cervical cancer vaccine,” follows this patriarchal value, impacting the victimization of women in HPV prevention. Under the patriarchal value system, the power relationship between men and women is inlaid and embedded into the configuration of HPV vaccine promotion that presents women as being vulnerable to HPV but disregards that HPV can cause any possible harm to men. Echoed with the situation in the United States [16], the promotion of HPV vaccine has pervasive gender bias as demonstrated from our findings. The HPV discourse focusing on women reinforces the patriachal belief that women are responsible for reproductive health in heterosexual relationship [42, 46]. The feminization of HPV vaccines is thus an amalgam of the scientific truth about the efficacy of the vaccine against cervical cancer and the embedded cultural narrative that women are “diseased bodies” and are more victimized in healthcare settings.

Feminization of HPV vaccines is also seen in the United States and causes different negative consequences to health promotion for both women and men, resulting in overburdening of women for screening and treatment of HPV-associated conditions and reduced protection from HPV-related illnesses for men [16]. In our study in Hong Kong, feminization of HPV vaccines also resulted in unintended consequences. In terms of vaccine hesitancy, feminization of HPV vaccines establishes a straightforward yet a simplified association between cervical cancer and HPV vaccination. While HPV vaccine is represented as highly efficacious to women, it also simplifies the risk of HPV merely with cervical cancer. This not only imposes the burden of disease on women but also misleads the risk assessment of HPV. As evident from our findings, the risk of cervical cancer was the only perceived risk of HPV, which served as an important factor of consideration in their decision-making process of vaccination. The risk of other but more common HPV consequences, such as genital warts, did not influence their decision-making process.

### Moralization of HPV vaccine

The complex moral implication of HPV vaccine served as another key determinant affecting the participants’ decision-making process. Following the argument of Sapir–Whorf hypothesis, the confusion between HPV and HIV led participants to have a stereotypical perception about the interrelationship between HPV, AIDS, cervical cancer, and immoral sexual activities. Such a perception served as an influential element in the decision-making process regarding HPV (non) vaccination, particularly for those were not vaccinated.

HPV was perceived by our participants to be sexually transmitted. Cervical cancer was consequently stereotyped as a condition mostly affecting sexually active and promiscuous women. HPV vaccine, thus, was perceived to be most needed by women who engaged in promiscuous and frequent sexual activities, serving as a subtle encouragement for having casual and immoral sexual behaviour, both of which are regarded as cultural taboos in Hong Kong. This stereotype contributed to a low perceived risk among participants due to their perceived moral sexual behaviour, which thereby led to their low perceived needs of getting vaccinated.

Advice from medical institutions embedded in the patriarchal value system also played a remarkable role in influencing the reputation of HPV vaccines. The vaccine has been presented as having the strongest efficacy for women without any sexual experiences [12]. In our participants, this accentuation led the development of a strong stereotype that only women with no sexual experiences qualify for getting vaccinated. HPV vaccine, at this point, has been associated with the moral values of women, making it appear that the vaccine is solely for sexually moral or abstinent women. Such accentuation fulfills and follows the cultural values and expectations for women in Hong Kong. HPV vaccine, thus, has a symbolic meaning and has been culturally constructed to become an indicator of sexual morals of women. Women fulfilling and following the cultural expectations could be considered as being more eligible than those offending them for getting vaccinated. On the other hand, participants did not feel motivated to receive HPV vaccination, as they perceived themselves to be sexually moral or abstinent on one hand, and HPV vaccination conveys a subtle symbol for casual and immoral sexual behaviour on the other. This contradicting perception served as a remarkable deterrent in their decision-making processes, limited their (social) accessibility to vaccination, and resulted in health inequality in terms of the undertaking of preventive health behaviour.

Such accentuation demonstrated the contradicting symbolic meaning of HPV vaccine. The power of cultural stereotype and stigma associated with HPV vaccination prevented participants from getting vaccinated, serving as a social control for women. Women are not only being culturally controlled for their (sexual) behaviours but also being deprived of having the rights to proactively protect their health, leading to their limited accessibility and health inequality in pursuing this preventive health behaviour. The cultural stereotype and stigma associated with HPV vaccination were remarkable elements that influenced the decision-making process, particularly in case of non-vaccinated participants.

### HPV vaccine hesitancy and distrust on private healthcare in Hong Kong

Participants were also reluctant to receive HPV vaccination considering the weaknesses of the macro-healthcare system and problematic doctor–patient relationship, which in turn affected their decision-making process. A recent study [23] investigated factors influencing vaccine acceptance in Botswana, the Dominican Republic, and Greece and reported that the operation of the public and private healthcare potentially leads to (dis) trust regarding vaccines among people. This finding concurs with our observations. Based on the comments of participants about HPV vaccine promotion and their experiences of interacting with doctors, we noted that healthcare in Hong Kong is also an underlying and inadvertent factor rendering the perception of people regarding HPV vaccines.

The healthcare system of Hong Kong follows a “dual-track system,” encompassing both the public and private sectors. While the former provides a “safety net for the entire community,” the latter offers “personalized choices and more accessible services to those who are willing and may afford to pay for private healthcare services” [24]. Such a system can provide basic healthcare service through the public healthcare system, usually to the lower social class, and can simultaneously provide private healthcare services to those who can afford paying more (usually the upper-middle and upper classes). On the other hand, scholars contend that the combination of the private and public healthcare sectors in Hong Kong is a “mixed-economy” system [43]. Although public healthcare service ensures that all Hong Kong citizens can enjoy basic medical service, it is mostly for secondary and tertiary care. The primary healthcare service, including vaccination, is mostly allotted to the private sector [13]. This practice of the healthcare system is a realization of capitalized logic—it is a way to outsource medical service to the private sector. In this manner, government expenditure can be reduced as the responsibility of providing primary care is passed on to the private sector.

The healthcare structure of Hong Kong driven by “mixed-economy” and capitalism plays a remarkable role in influencing the perception of people regarding doctors, restraining the acquisition of information on vaccination and impacting their trust on doctors and the vaccine. There reportedly exists an embedded distrust on the private healthcare system and private practice doctors [43]; we also observed this. As evident via our observations, if doctors suggested vaccination, such a suggestion was very often interpreted with suspicion; participants believed that doctors had commercial interests by “selling” the vaccine and were trying to earn profit. Moreover, participants perceived private practice doctors as having a stereotypical relationship with business and profit-making. These stereotypes undermined their trust on private practice doctors, making participants suspicious of every suggestion made by doctors, including that to get vaccinated. This embedded distrust on private healthcare could extend the distrust on HPV vaccines, particularly because the vaccine is solely available via the private healthcare section for most people in Hong Kong at the study time. The commercial stereotype attached on private practice doctors influenced the perceived creditability of the information and understanding of the needs and efficacy of HPV vaccine. This embedded stereotype of private healthcare can also explain why healthcare providers were not enthusiastic to provide information regarding HPV vaccination to participants. This inaccessibility of information led to lack of awareness, understanding, and positive influence of the vaccine among participants. As HPV vaccination, if not under the government-funded Community Care Fund Cervical Cancer Vaccination Pilot Scheme for low-income adolescent girls, is available mostly via the private healthcare system, distrust on private healthcare is bound to influence the decision-making process.

The lack of the family doctor system in Hong Kong is also responsible for the lack of trust between doctors and patients, making preventive care at the primary care level, such as advice regarding vaccination, difficult. As majority of the primary care service is provided by the private healthcare system in Hong Kong [13], distrust on private practice doctors along with lack of the family doctor system has contributed to a weak doctor–patient relationship. In the absence of trust between doctors and patients, doctors can feel hesitant to suggest vaccination, which is an additional service that does not fit within the clinical norms in Hong Kong, though preventive care is an important element of primary care [55]. Healthcare practice in Hong Kong mainly focuses on curative treatment. Such a clinical norm hindered participants from obtaining preventive care, such as HPV vaccine-related information. As there is no intention of offering preventive treatment, discussions regarding vaccination have ceased to be a part of the consultation process with doctors. On the other hand, HPV vaccine is not included in the government’s immunization program in Hong Kong. There are only irregular school-based subsidized HPV vaccination programs for students in addition to a Community Care Fund Free Cervical Cancer Vaccination Pilot Scheme that financially supports females who are aged 9–18 years and belong to low-income families to receive HPV vaccines. Those who do not fall under these categories can only take the vaccine through private practice clinics and hospitals on a self-pay basis. This results in an inequity of accessing vaccination as preventive health behaviour. The macro-healthcare system of Hong Kong, thus, serves to create HPV vaccine access inequality to some extent; HPV vaccine inaccessibility influenced the decision-making process of our study participants. As most primary care doctors in Hong Kong are a part of private practice, relying upon them for vaccination may not be feasible considering that they have been negatively stereotyped in Hong Kong. Rather, the government health authorities should take more proactive action in promoting HPV vaccination considering the trust from public.

Our participants widely perceived government health authorities to be the most trustable and credible sources of information on HPV vaccines. This finding was consistent with the past literature, indicating that people in Hong Kong have a higher trust on public than on private healthcare as there is no association between public healthcare and business [43]. However, participants encountered remarkable difficulty in obtaining information regarding HPV vaccines from government health authorities. Without the perceived trustable and credible information from the government, participants turned to believe that HPV vaccine is not an important healthcare measure. Social authority, such as government institutions, can create social belief and norms [56]. This lack of information led to HPV vaccination being perceived as an unimportant preventive healthcare measure. As stated earlier, the suggestion of private practice doctors to get vaccinated was interpreted as being unnecessary and treated with suspicion, and this belief was further reinforced by mass media and the Internet reporting about the side-effects of the HPV vaccine, causing even more negativity regarding the vaccine among participants. Participants perceived news reports and the Internet as relatively more credible sources of information than private practice doctors, strengthening their distrust on private healthcare and private practice doctors. This consequently negatively influenced the decision-making process of participants.

### New discovery suggested for the vaccine hesitancy matrix

Our findings indicate that the decision-making process regarding whether to receive HPV vaccination is a complex one, involving interlinked factors. Based on the education level and socioeconomic status of participants, financial factors were a less important determinant affecting the decision-making process. However, this does not imply that vaccine cost was irrelevant; the decision of HPV vaccination was affected by the assessment of worthiness of the vaccine, which in turn was affected by diverse perceptual, social, cultural, and structural factors, rather than simply the monetary cost.

Our study findings also somewhat consistent with the concept of vaccine hesitancy [39], as we report that several determinants in particular impact participants. These include communication and media, personal experiences and those of significant others, perceptions of the healthcare system, and perceived risk of HPV. However, we also report that there are other determinants that are not mentioned in the vaccine hesitancy determinant matrix. Cultural determinants such as experiences of sexual activities, social stigma associated with HPV vaccination, presence (or absence) of perceived trustable information, and perception of HPV vaccines were all remarkable in case of our participants. Furthermore, structural factors also matter—in the absence of trust on private practice doctors, participants perceived their advice regarding vaccination with suspicion, irrespective of how enthusiastic doctors were to give such an advice. Our findings also showed some consistency with the three Cs model of vaccine hesitancy [39, 50], although we observed that confidence and complacency were comparatively more influential than convenience.

### Limitations

As we mostly sampled women from a local university and a primary care clinic located in a middle-income district with a relatively high percentage of population with postsecondary education, hence our findings mostly reflect the perceptions and decision-making process of women who belong to a relatively high socioeconomic status. A more holistic view can be achieved by sampling women belonging to a low socioeconomic status and from different field sites. However, our sampling type allowed us to gain an in-depth understanding of the complex perceptual, social, and cultural issues that influence the decision-making process in women, apart from the financial concerns related to HPV vaccination.

## Conclusions

This study, from perceptual, social, cultural, and structural aspects, discusses the interlinked factors that influence the decision-making process regarding HPV vaccination in women in Hong Kong. The promotion of HPV vaccination is “feminized” and “moralized,” which consequently influenced the understanding of our study participants regarding HPV vaccination, and limited their accessibility of vaccination as preventive health behaviour. Health inequality in accessing HPV vaccine has thus been resulted. The “mixed-economy” medical system of Hong Kong driven by capitalist logic also makes preventive care absent in the consultation process between patients and doctors, molding a weak doctor–patient relationship, making the implementation of advice from private practice doctors regarding vaccination even more difficult. The government health authorities, therefore, should take more proactive action in promoting HPV vaccination considering the trust from public.

## Data Availability

The datasets generated and/or analysed during the current study are not publicly available due to protection of participants’ confidentiality, but are available from the corresponding author on reasonable request.

## Appendix

### Interview question guide

A. Participants’ knowledge and risk perception of HPV

1. What is your impression of HPV?
2. How do you perceive the dangers of HPV?
3. What can be the worst consequence(s) if having HPV?
4. What do you think about your risk of getting HPV? Why?
5. Are there any people whom you think the most vulnerable of getting HPV?

B. Participants’ knowledge, acceptance, and perception of HPV vaccines

1. From what sources have you heard about HPV and HPV vaccines?
2. Can these sources motivate and/or discourage you to consider receiving HPV vaccination? Why? (relevant to F1, F2)
3. What is your impression of HPV vaccines? What are they for?
4. What do you think about your need for getting vaccinated? Why? (relevant to F1, F2)

C. Influence from social norms and participants’ significant others on HPV vaccination incentives and barriers

1. Is there any discussion about HPV and HPV vaccines in your social sphere?
2. Who has ever talked about HPV and HPV vaccines in your social sphere? Who has talked the most?
3. Who has never talked about HPV and HPV vaccines in your social sphere?
4. Can these people motivate and/or discourage you to receive HPV vaccine? Why? (relevant to F1, F2)
5. What do you think about the social atmosphere of Hong Kong with regard to HPV vaccination?
6. In your opinion, how do society members view HPV vaccination?
7. In your opinion, how do society members view cervical cancer (for both sexes), penile cancer (for males) and warts (for both sexes)?
8. Can the social atmosphere and social members motivate and/or discourage you to receive HPV vaccine? Why? (relevant to F1, F2)

D. Influence from health care providers on HPV vaccination incentives and barriers

1. Do you have any family doctor, or any doctor(s) whom you always see?
2. Have you ever considered about receiving the vaccine / asking about the vaccine information from your family doctor / usual doctor? Why?
3. If you have, how is your experience? How did your doctor respond to your question?
4. Did the doctors’ response / attitude affect your motivations in getting vaccinated? (relevant to F1, F2)

E. Perceptions of suitable social groups in receiving HPV vaccinations

1. Who should receive HPV vaccines? Why? (Probe: “Who” in terms of occupation, generation, sex, age …)
2. What age do you think as the suitable age of receiving HPV vaccine? Why?
3. Will you consider HPV vaccine is suitable for you and for your children? Why? (Probe the ages of their children)

F. Participants’ incentives of and barriers to receiving HPV vaccines

1. What can motivate you to consider receiving HPV vaccination?
2. What can discourage you from considering to receive HPV vaccination?

G. Perceived benefits and negative influences of receiving HPV vaccinations

1. How do you think about the benefits of receiving HPV vaccine? (Probe in terms of physical health, social, and cultural impacts …)
2. How do you think about the negative influences of receiving HPV vaccines? (Probe in terms of physical health, social, and cultural impacts …)

H. Demographics

1. Sex
2. Age
3. Marital status
4. Any daughters / sons?
5. Age of daughters / sons
6. Education level
7. Occupation
8. Income level
9. Ever received HPV vaccination?
10. Any people you know have ever received HPV vaccination?
11. Ever received HPV screening / cervical screening (Pap Smears)?

## Abbreviations

AIDS: Acquired immune deficiency syndrome
HIV: Human immuno-deficiency virus
HPV: Human papillomavirus
